# The connection between professional burnout of medical workers and the self-help methods during the COVID-19 pandemic

**DOI:** 10.1192/j.eurpsy.2024.1059

**Published:** 2024-08-27

**Authors:** E. V. Deshchenko, J. E. Koniukhovskaia, O. B. Stepanova, I. M. Shishkova, E. I. Pervichko, O. V. Mitina, N. R. Irgashev

**Affiliations:** ^1^ Lomonosov Moscow State University; ^2^Higher School of Economics, Moscow; ^3^Ryazan State Medical University, Ryazan, Russian Federation

## Abstract

**Introduction:**

Many medical workers suffered from severe professional burnout while working in the conditions of the COVID-19 pandemic, but few of them had the opportunity to find psychological help.

**Objectives:**

The aim of the research was to study the relationship between emotional burnout and self-help strategies in medical professionals during the pandemic.

**Methods:**

The Maslach Burnout Inventory (MBI) was used to measure the level of professional burnout. It was filled out by medical workers from January 2021 to November 2022.

The sample consisted of 314 medical workers (57 men and 255 women), whose average age was 36.97±11.93. According to the level of education, the sample included specialists with secondary general education (4.14%), with secondary special education (19.4%), with incomplete higher education (11.46%), with higher education (59.87%) and PhD (5.1%). 35 people (11%) of the surveyed medical workers worked in the red zone.

**Results:**

When medical workers experience severe Emotional Exhaustion and Depersonalization, they often try to help themselves by drinking alcohol (r=0.156; p=0.005; r=0.184; p=0.001), eating (r=0.227; p=0.000; r=0.151; p=0.007), taking medications (r=0.204; p=0.000; r=0.212; p=0.005), solitude (r=0.204; p=0.000; r=0.133; p=0.019), watching TV series (r=0.173; p=0.002; r=0.146; p=0.01). With an increase in the Reduction of professional skills, medical workers also eat more (r=-0.148; p=0.009) and try to learn something new, engage in self-development (r=-0.137; p=0.015). It is important to note that the desire to seek psychological help is associated only with Emotional Exhaustion (r=0.121, p=0.032), that is, he/she may be aware at an early stage of professional burnout, when the symptoms of depersonalization and reduction of professional skills have not yet occurred.

**Conclusions:**

Thus, all the considered self-help methods are already used with pronounced symptoms of professional burnout, but do not lead to its pronounced decrease. It is important to note that seeking psychological help is possible with awareness of emotional exhaustion, but not with depersonalization and reduction of professional skills.

Disclosure: Research is supported by the Russian Science Foundation, project No. 21-18-00624.

**Disclosure of Interest:**

None Declared

